# A Self-Centering, Blade-Assisted, Electrowetting-Enabled Strategy for Precise Droplet Splitting on Open Digital Microfluidic Platforms

**DOI:** 10.3390/mi17070839

**Published:** 2026-07-15

**Authors:** Hao Liang, Liang Chen, Haifeng Zhang, Xiaowei Liu

**Affiliations:** School of Astronautics, Harbin Institute of Technology, Harbin 150001, China; lianghao_edu@163.com (H.L.); cliang@hit.edu.cn (L.C.); zhanghf@hit.edu.cn (H.Z.)

**Keywords:** droplet microfluidic, EWOD, droplet splitting, open DMF platforms

## Abstract

Droplet splitting technology on open digital microfluidic platforms still faces significant challenges in terms of process complexity, the degree of automation, and operating conditions, which hinder its further development. This study proposes a fully automated method for precise droplet splitting based on printed circuit boards with open-coplanar asymmetric electrodes and a slippery liquid-infused porous surface. This method uses simple square electrodes arranged in a 3 × 5 array, combined with low-adhesion blade-assisted cutting and electrowetting-on-dielectric to drive droplet splitting, enabling accurate, stable, and repeatable automated droplet splitting on an open digital microfluidic platform. It has the advantages of a simple method, easy maintenance and integration, and high automation. This study systematically investigated the effects of droplet volume, applied voltage, blade thickness, cutting speed, and electrode shape on droplet splitting performance. We developed an active droplet position calibration method based on a simple 3 × 3 square electrode array combined with an enveloping voltage configuration strategy. For droplets with a volume of 10 μL, the positioning error can be controlled to within 0.06 mm, representing a reduction of more than 95% compared to the conventional EWOD free drive method. The experimental results show that to achieve stable and approximately equal-volume droplet splitting, the cutting speed needs to exceed the critical value related to the blade thickness. Among the square, zigzag, and hexagonal electrode shapes tested, the square electrode required the lowest splitting voltage. When the blade thickness is 0.3 mm, the droplets can be successfully split at a minimum voltage of 165 V. After increasing the splitting voltage to 400 V, the droplet splitting time was reduced from 5.57 s to 0.27 s, with a reduction of 95.2%, which significantly improves droplet splitting efficiency. This method provides a practical, stable, automated, and precise droplet splitting method for sample preparation, biochemical reactions, and portable droplet analysis systems.

## 1. Introduction

The PCB-SLIPS open digital microfluidics (DMF) platform, fabricated using multilayer printed circuit board (PCB) technology and a slippery liquid-infused porous surface (SLIPS), has been widely applied in large-scale electrowetting-on-dielectric (EWOD) DMF devices due to its advantages such as flexible sample operation, simple structure, resistance to electrical breakdown, low bio-adhesion, low contact angle hysteresis, low cost and high integration [1,2,3,4]. On open DMF platforms, manipulation techniques such as droplet generation [5], transport [6,7], and mixing [8] have been widely studied. However, compared with the mature droplet splitting techniques [9,10,11,12,13] on closed DMF platforms, there are currently no simple and effective solutions for droplet splitting on open DMF platforms. This has significantly hindered the further development of open DMF platforms. Up to now, the droplet splitting in an open environment has been studied by some scholars. For example, Bormashenko et al. used a superhydrophobic scalpel on a superhydrophobic surface to cut a droplet into two equal parts at a high cutting speed [14]. Yanashima et al. achieved droplet cutting on a superhydrophobic surface by using a superhydrophobic blade in conjunction with the stretching of metal rings on both sides [15]. Kiani et al. stretched droplets on a superhydrophobic surface using needles placed vertically and horizontally, and then cut the droplets with the movement of a superhydrophobic blade in the vertical direction [16]. Han et al. proposed a method for noncontact manipulation of liquids under voltages of several thousand volts using a charge-shielding mechanism [17]. Sagar et al. used composite droplets to achieve droplet splitting on an open EWOD platform [18]. Chen et al. proposed a method that combines surface acoustic wave (SAW) and EWOD techniques to achieve particle concentration regulation within droplets and droplet splitting on coplanar interdigitated electrodes [19]. However, all these methods generally have problems such as complex surface treatment processes, manual operations, high-voltage conditions, special electrode patterns, and difficulties in droplet collection. They cannot effectively solve the key technical problems in the automated droplet splitting on open DMF platforms. Therefore, we propose a fully automated, precise droplet splitting method for the open PCB-SLIPS DMF platform in combination with low-adhesion blade-assisted cutting. Using simple square electrodes arranged in a 3 × 5 array, a fully automated droplet splitting process is achieved through three simple and controllable steps: active droplet centering, low-adhesion blade-assisted cutting, and EWOD-assisted droplet splitting. The influencing factors of droplet self-centering position deviation and the influence mechanisms of blade thickness, cutting speed, applied voltage, and electrode shape on droplet splitting performance were systematically studied. Compared with the existing open platform droplet splitting methods, the droplet splitting strategy proposed in this paper can achieve the autonomous centering and positioning of droplets. Combined with EWOD technology, it effectively eliminates the cumbersome manual use of external auxiliary devices and has a high degree of automation. It avoids the drawbacks associated with the oil–water composite droplets required in the open DMF platform, and the droplet collection and post-processing methods are simple; the operating voltage is far lower than the several thousand volts required for a charge-shielded knife, placing fewer demands on the system; the simple PCB electrode array is used, which has the advantages of universality, economy and integration compared with the SAW-EWOD hybrid structure. This method is expected to provide a fast, accurate, and fully automated droplet splitting strategy for the new open DMF platform.

## 2. Experimental Section

### 2.1. Experimental Setup and Method

**Figure 1:**
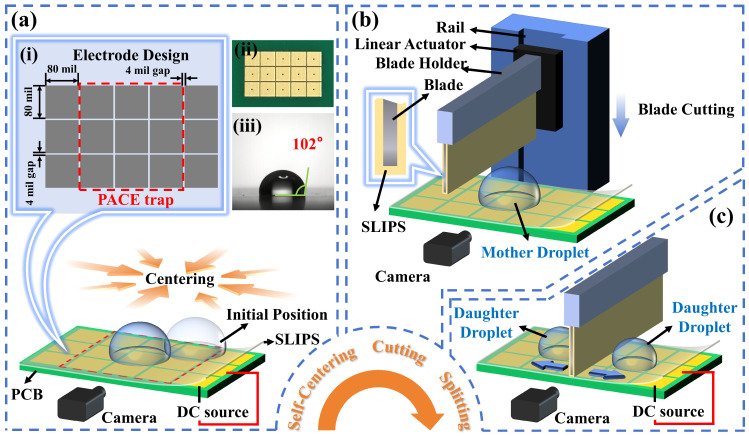
The process of the droplet splitting: (**a**) self-centering. (**i**) schematic diagram of the electrode’s geometric design and dimensional parameters; (**ii**) photograph of the electrode; (**iii**) contact angle of the PCB-SLIPS; (**b**) cutting. (**c**) splitting.

The automated droplet splitting process mainly consists of three steps: self-centering, cutting, and splitting (Figure 1). The experimental setup of this process mainly consists of two parts: the PCB-SLIPS chip and the cutting unit (Figure 1a,b). The PCB-SLIPS chip consists of a PCB substrate with a 3 × 5 coplanar electrode array unit structure (Figure 1a(ii)) fabricated by commercial PCB technology and a poly-tetrafluoroethylene (PTFE) porous membrane filled with silicone oil laid flat on the electrodes (Figure 1a). The DC voltage is applied to the corresponding electrode in accordance with the design logic by using sequential signals to control the relay. The cutting unit consists of a cutting blade, holder, rail, and linear actuator. The cutting blade is fixed on the linear actuator by the holder. The linear actuator can move up and down along the rail, which drives the cutting blade to achieve precise, speed-controllable, and repetitive motion in the vertical direction. The cutting blade is made of a metal material. The cutting blade is coated with a PTFE porous membrane prepared by the same process as the PCB-SLIPS chip.

During the self-centering process, the fully automated droplet segmentation strategy proposed in this paper is based on an open digital microfluidic platform. In this strategy, droplets are loaded by using the EWOD driving technology on the open DMF platform to automatically guide them into the electrode region. Subsequently, the active centering of the droplet is achieved by using a simple 3 × 3 square electrode array in combination with the enveloping voltage configuration strategy (Figure 1a(i)). During the cutting process (Figure 1b), first, the optical marking combined with the fine-tuning platform is used to align the center of the blade with the centerline of the center electrode at the same time. Then, the linear actuator is used to precisely control the falling stop position of the blade and stop when it just touches the PTFE membrane on the PCB-SLIPS chip. This ensures that the blade completely cuts through the mother droplet while also preventing damage to the SLIPS coating on the PCB and the cutting blade, resulting in high reliability and durability. During the splitting process (Figure 1c), the EWOD force is used to split the two daughter droplets from Blade-SLIPS, ultimately achieving successful droplet splitting. The droplet splitting process is recorded by a CCD camera through frontal observation (Figure 1). The complete automatic splitting process of the 10 µL droplet is shown in Video S1, including droplet self-centering, cutting blade descent-cutting-stopping, EWOD-assisted detachment, blade recovery, and the immediate distribution of the daughter droplets after cutting.

### 2.2. Fabrication of the PCB-SLIPS Chip

The electrodes of the PCB-SLIPS chip are a 3 × 5 array unit. The electrode shape is square, and its size is 80 mil × 80 mil. The distance between two adjacent electrodes is 4 mil, and the gap depth is approximately 40 μm. The surface roughness of the electrode and the oxide layer has a significant impact on the droplet movement. In this study, gold plating (2U″) treatment was carried out on the copper electrode surface of the PCB to reduce the surface roughness of the electrode and act as an anti-oxidation layer. The photograph of the electrode is shown in Figure 1a(ii). We used the central 3 × 3 electrode array combined with an enveloping voltage configuration strategy to achieve active position calibration of the droplets. This 3 × 3 electrode array unit is named as peripheral-actuated central-grounded EWOD centering trap (PACE trap) (Figure 1a(i)). On the modified SLIPSs, droplets can move between two separate square electrodes, and they tend to move from the electrode with a higher potential to the one with a lower potential [20,21,22]. According to the research of Al-Lababidi et al., the droplet has the greatest EWOD driving force under the electrode configuration of “all ground” [23,24,25]. Therefore, the 3 × 5 electrode array unit in this study not only ensures the self-centering position calibration operation of the droplet but also provides the required symmetrical electric field geometry for reliable droplet splitting after cutting. The 3 × 5 electrode array unit can provide grounded target electrodes for the daughter droplets during droplet splitting and arrange high-potential electrodes around them in a symmetrical electrode configuration operation. The daughter droplets on both sides of the blade are pulled from the high potential area near the blade towards the grounded target electrode. This design enhances the horizontal EWOD force component along the splitting direction and suppresses the undesired movement of daughter droplets away from the target electrode. It has the advantages of a small number of electrodes, easy wiring, and integration.

For the preparation of PCB-SLIPS, first, the PTFE porous membrane was cut according to the size of the electrode array. Then it was immersed in 0.5 wt% trimethoxy (1H, 1H, 2H) 2H-heptadecafluorodecyl) silane ethanol solution at room temperature for 1 h to obtain a modified PTFE porous membrane. It was then dried at 120 °C for 2 h to remove the excess fluorinating agent. Next, the modified PTFE membrane was placed over the clean PCB electrode substrate and stretched slightly to make it flat. Finally, the PMX-200 polydimethylsiloxane with a viscosity of 1 cSt was infused into the modified PTFE porous membrane by capillarity to form a transparent SLIPS. The sample was placed vertically for 1 h to remove excess PMX-200 polydimethylsiloxane and then placed horizontally for 1 h to obtain a lubricating layer of uniform thickness. So far, the PCB-SLIPS has been fabricated. The contact angle of the PCB-SLIPS is 102° (Figure 1a(iii)).

### 2.3. Fabrication of the Cutting Unit

The cross-section of the blade in the cutting unit is rectangular, and the bottom is planar (see the enlarged view of the blade in Figure 1b). Compared to blades with sharp-edged structures, this method has a simple structure and is easy to apply in engineering. In this study, the size of the blade is length × width = 20 mm × 20 mm, and the thicknesses are 0.1 mm, 0.2 mm, 0.3 mm, 0.4 mm, 0.5 mm, and 1 mm. The blade material is selected as aluminum alloy, which is resistant to deformation and is lightweight. It can ensure high repeatability and stability during the droplet cutting process, has low requirements for the system, and is easy to integrate. We coated the bottom and both sides of the blade with a PTFE porous membrane using the same preparation process as in Section 2.2 to prepare Blade-SLIPS on the blade to reduce the viscous resistance on the blade surface during droplet splitting (Figure 1b). Compared to other surface treatment methods for blades [16,26], this method does not require complex processes and is simple to implement. Furthermore, during the usage process, the surface properties can be easily and quickly restored by adding PMX-200 polydimethylsiloxane or directly replacing the newly prepared PTFE porous membrane, which is easy for engineering applications.

### 2.4. Materials

The material of the blade is aluminum alloy (model: 1060, 20 mm × 20 mm), purchased from Zhejiang Hongnian Aluminum Industry Co., Ltd., Zhejiang, China. A standard two-layer PCB with EWOD electrodes was fabricated on rigid Flame Retardant 4 (FR4) material using commercially available PCB technology (Shenzhen JDB Technology Co., Ltd., Shenzhen, China). The PTFE porous membrane (composed of nanofibrous networks, ~35 μm thick, with an average pore size of 220 nm) was purchased from Suzhou Unique New Material Sci. & Tech. Co., Ltd., Suzhou, China. Anhydrous ethanol and trimethoxy (1H, 1H, 2H, 2H-heptadecafluorodecyl) silane were purchased from Shanghai Aladdin Biochemical Technology Co., Ltd., Shanghai, China. PMX-200 polydimethylsiloxane with a viscosity of 1 cSt was obtained from Dow Corning, Midland, MI, USA. The liquid sample used in this study is deionized water (resistivity: 17 MΩ·cm), which was prepared using a laboratory water purification system from Harbin Yinghua Water Industrial Technology Development Co., Ltd., Harbin, China.

### 2.5. Instruments and Characterizations

The contact angles (CAs) of 5 μL deionized water were measured by a contact angle meter system (JC2000D2A, Shanghai Zhongchen Digital Technic Apparatus Co., Ltd., Shanghai, China) at room temperature. The CA values are the average of five measurements. The droplet splitting process was recorded with a CCD camera (Lumenera Lu135, Waterloo, Canada). The DC voltage is provided by a high-voltage DC power supply (DW-P162-300AC, Dongwen High Voltage Power Supply (Tianjin) Co., Ltd., Tianjin, China). The linear actuator (CTM28) was purchased from Beijing Haijie Jachuang Technology Co., Ltd. (Beijing, China). It has a positioning accuracy of ±5 μm and an adjustable speed range of 0–100 mm/s. The relays were purchased from Shenzhen Kobe Electronic Technology Co., Ltd. (Shenzhen, China).

## 3. Results and Discussion

### 3.1. Mechanism of Droplet Splitting

**Figure 2:**
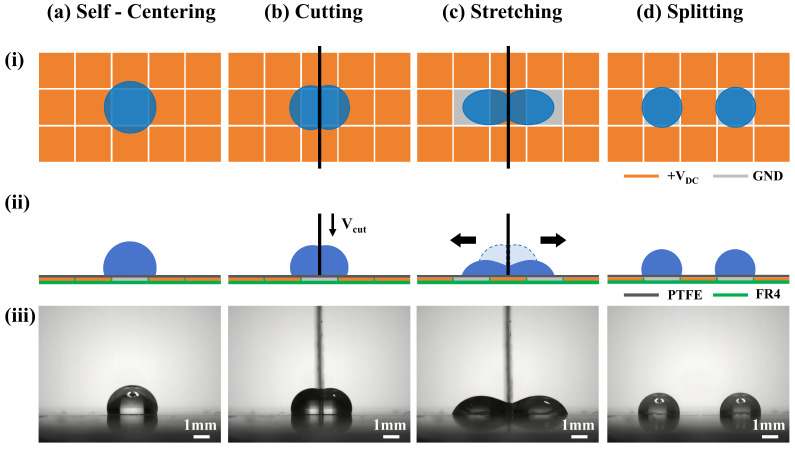
Schematic diagram of the droplet splitting mechanism. The droplet splitting process can be divided into four continuous stages: (**a**) self-centering; (**b**) cutting; (**c**) stretching; (**d**) splitting. Among them, (**i**) the top view of the droplet splitting mechanism; (**ii**) the front view of the splitting mechanism; (**iii**) the experimental result of the corresponding stage of droplet splitting with a blade thickness of 0.1 mm.

The droplet splitting mechanism is shown in Figure 2. The entire process can be divided into four continuous stages: self-centering, cutting, stretching, and splitting. A DC voltage signal is used to drive the droplets. The voltage loading and polarity transformation on the electrode are controlled by the relay periodically controlled by the microcontroller to achieve droplet splitting. In this study, the initial position of the mother droplet does not need to be precisely controlled. It only needs to be within the 3 × 3 square electrode area of the PACE trap. First, using the PACE trap with an electrode configuration in which +V_DC_ is applied to the peripheral electrodes and the central electrode is grounded, the mother droplet is self-centered and positioned at the center of the electrode array (Figure 2a). Then, adjust the cutting blade to the centerline position of the central electrode. The linear actuator is then controlled to drive the blade to cut the mother droplet vertically at a certain speed V_CUT_, dividing the mother droplet into two daughter droplets of equal volume, symmetrically distributed on both sides of the cutting blade (Figure 2b). Next, change the polarity of the central electrode and the adjacent two electrodes. Transform the central electrode to apply +V_DC_ and the adjacent two electrodes to ground. Meanwhile, +V_DC_ is applied to all other electrodes in the 3 × 5 electrode array unit (Figure 2c). This electrode configuration not only ensures a strong electric field but also effectively restricts the horizontal movement of the daughter droplets, thereby improving the splitting efficiency. Using the principle of polarity-dependent electrowetting, the daughter droplets on both sides of the blade are stretched outward (Figure 2c). The droplet splitting process is complete once the daughter droplets have detached from the Blade-SLIPS (Figure 2d).

### 3.2. Self-Centering Performance of the PACE Trap

**Figure 3:**
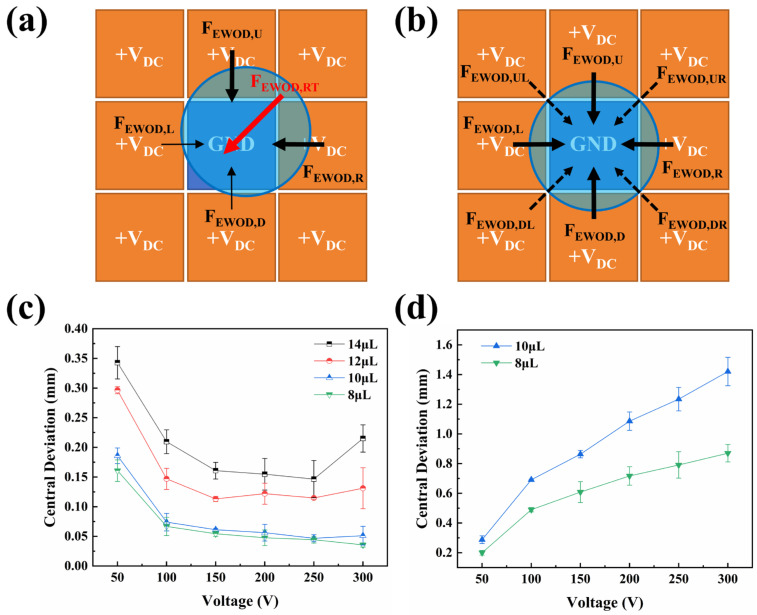
Self-centering mechanism and performance characterization of the PACE trap. (**a**) Schematic illustration of the force exerted by the PACE trap to drive the droplet toward the center when it deviates from the center; (**b**) schematic illustration of the forces acting on the centered droplet; (**c**) central deviation of droplets with different volumes under different applied voltages using the PACE trap; (**d**) central deviation of droplets under EWOD free-drive mode without the PACE trap.

The initial position of the mother droplet plays a critical role in the quality and repeatability of the cut. In fact, it is very difficult to place the mother droplet completely at the center of the electrode array. The position of the mother droplet directly affects the initial cutting position of the cutting blade, which seriously influences the relative size of the daughter droplets after cutting. To improve the repeatability of droplet positioning and the accuracy of the initial position, this study used a simple 3 × 3 square electrode array unit, combined with a voltage configuration strategy in which the eight surrounding square electrodes were connected to +V_DC_ and the central electrode was grounded (GND). This formed a converging EWOD force field directed toward the center. This induced the droplets to move toward the central grounded electrode, achieving self-centering of the droplets (Figure 3a,b). This structure provides a simple and effective strategy for droplet self-centering on open DMF platforms.

The schematic diagram of the principle of implementing droplet self-centering in the PACE trap is shown in Figure 3a. When the center of mass of the droplet deviates from the geometric center of the central ground electrode, the overlapping region between the three-phase contact line of the droplet and the surrounding driving electrodes will become asymmetrical. Due to the polarity-dependent electrowetting effect, an EWOD resultant force (F_EWOD,RT_) directed toward the central grounded electrode is generated (Figure 3a), which drives the droplet toward the central grounded electrode. As the droplet gradually approaches the center position, the EWOD force components from the left (F_EWOD,L_), right (F_EWOD,R_), top (F_EWOD,U_), bottom (F_EWOD,D_), and diagonal directions gradually tend to balance out (Figure 3b). Therefore, the central position corresponds to a state of electric force equilibrium, in which the EWOD resultant force acting on the droplet in all directions is zero. This force-balancing mechanism makes the PACE trap different from the conventional EWOD free-drive method. By simply using a 3 × 3 electrode array unit, it converts the polarity-dependent EWOD driving force into a spatial centripetal force field, effectively suppressing the final position error of the droplet. In the free-drive mode, the final stopping position of the droplet is easily affected by factors such as inertia, deformation, and the pinning of the contact line, and may deviate randomly from the center of the grounding electrode.

To quantitatively evaluate the self-centering performance of the PACE trap, this study statistically analyzed the deviations between the center of mass of droplets of different volumes and the geometric center of the central electrode under different driving voltages. The results are shown in Figure 3c. After the self-centering process begins, wait 15 s to ensure that the droplet is fully centered. The position of the droplet center point and the midline position of the central electrode were found by ImageJ (2.18.0), and the distance between them was calculated to obtain the center deviation under the corresponding conditions. The droplet centering experiment under each condition was repeated five times, and the average value was taken as the result for that condition. The error bars in Figure 3c,d represent the standard deviations of the five groups of data. For small to medium volume droplets, especially 8 μL and 10 μL droplets, the PACE trap shows excellent self-centering ability within a wide voltage range. When the voltage increased from 100 V to 300 V, the center deviation for both 8 μL and 10 μL droplets remained below 0.075 mm, and at voltages above 150 V, it decreased to below 0.06 mm. This deviation accounts for only about 3% of the electrode width (80 mil), indicating that the droplets can be precisely confined to the central region of the 3 × 3 electrode array unit. In contrast, larger droplets exhibit relatively weaker centering performance. The 12 μL droplets showed a moderate degree of centering deviation, while the 14 μL droplets had the greatest deviation and were more sensitive to voltage changes. This phenomenon can be attributed to a mismatch between the droplet volume and the geometric size of the PACE trap. For larger droplets, the bottom contour of the droplet overlaps with multiple surrounding electrodes over a wider area, thereby reducing the geometric symmetry of the EWOD forces acting on the droplet and increasing its deformation during the driving process. Consequently, the stability of the force balance at the center is reduced, resulting in an increase in the final offset. These results indicate that the self-centering performance of the PACE trap is closely related to the match between droplet volume and electrode spacing. In addition, overly small droplets are not conducive to automated transport on the open DMF platform. According to our experimental measurements, the diameter of a 4 μL droplet on PCB-SLIPS is 2.184 mm, slightly larger than the width of the current electrode (80 mil). Therefore, droplets smaller than 8 μL are not suitable for droplet splitting operations under the current electrode size design. Therefore, for the current 80 mil electrode design, 8–10 μL droplets are more suitable for achieving stable self-centering.

Figure 3d shows the deviation of 8–10 μL droplets from the geometric center in the EWOD free-drive mode. By comparison, it can be found that in the free-drive mode, although the droplet can stop on the ground electrode after being driven, the droplet is not constrained by the force field pointing towards the center. Therefore, the droplet center deviation will increase significantly with the rise in the driving voltage. For a 10 μL droplet, the deviation increased from 0.29 mm at 50 V to over 1.42 mm at 300 V. The 8 μL droplet also exhibited a similar trend, with its deviation increasing from approximately 0.20 mm to 0.87 mm within the same voltage range. The main reason for the increase in deviation under free-drive mode is that high voltage will increase droplet velocity, resulting in more obvious droplet deformation and inertial motion. Compared to free-drive mode, the PACE trap significantly reduces the error in the droplet’s final position. At 300 V, for a 10 μL droplet, the PACE trap reduced the center deviation from 1.42 mm in the free-drive mode to 0.05 mm, with a deviation reduction of 96.5%. For an 8 μL droplet, the center deviation also decreased from 0.87 mm to 0.04 mm, representing a 95.4% reduction in deviation. These results demonstrate that the PACE trap can effectively correct the final position of a droplet through a symmetric centripetal EWOD force field, which is of great significance for the accuracy, reliability, and automation level of the subsequent droplet cutting operation.

Based on the above results, although an 8 μL droplet also exhibits good centering performance, a 10 μL droplet can provide a larger liquid volume for subsequent microfluidic operations, which is beneficial for droplet merging, reactions, and detection. In addition, when the voltage is increased to 150 V, the center deviation of the 10 μL droplet has already decreased to a relatively low level (less than 0.06 mm). When the voltage is further increased, the positioning accuracy is not significantly improved. Instead, it may aggravate the droplet deformation and increase the electrical load of the system. Taking all the above factors into consideration, subsequent experiments were conducted using a droplet volume of 10 μL and a self-centering voltage of 150 V. This can not only ensure a good electrode size match but also provide a larger droplet volume and reliable positional accuracy, while maintaining stable droplet morphology and droplet control.

### 3.3. Effect of Blade Cutting Speed and Blade Thickness on Droplet Cutting

#### 3.3.1. Effect of Blade Cutting Speed on Droplet Cutting

The cutting of a droplet by a blade can be understood as a dynamic interfacial transformation process. During this process, the mother droplet, which originally had a continuous interface, is split by the blade into two separate daughter droplets. The blade imposes localized geometric constraints at the liquid–gas interface of the mother droplet and forces the liquid near the cutting plane to redistribute laterally on both sides of the blade. Therefore, whether a droplet can be successfully split in half depends on the competition between the interface splitting process induced by the blade and the droplet interface recovery process driven by capillary force.

When the blade meets the droplet, the liquid–gas interface at the top of the droplet will locally concave and form a high-curvature area near the edge of the blade. The resulting Laplace pressure gradient will drive the liquid to flow from the compressed central area to both sides of the blade [27,28]. As the blade continues to cut into the liquid, the volume of liquid in the center of the droplet is continuously pushed out, and two lateral liquid bulges gradually form on both sides of the blade. At this stage, the droplets remain connected to each other, and the liquid bodies on the left and right sides are linked by tiny liquid bridges near the blade and the substrate. The subsequent evolution of the liquid bridge determines the final cutting result. At a lower blade cutting speed (V_CUT_), the cutting process is close to a quasi-static deformation process. At this point, the liquid–gas interface has enough time to smoothly deform around the blade to reduce the surface energy of the system instead of causing fracture. At the same time, the liquid can flow along the side walls of the blade and redistribute itself around the blade. Under these conditions, the central liquid bridge is continuously replenished by capillary reflux, and the droplets tend to remain connected or migrate preferentially to one side of the blade. The result is manifested as incomplete cutting, asymmetric splitting, or remerging of the two liquid areas on one side of the blade. When the V_CUT_ of the blade exceeds a certain critical value, the cutting process enters a state of dynamic instability. At this point, the speed at which the blade penetrates the droplet is faster than the speed at which the liquid interface recovers. Therefore, before the capillary reflux reestablishes the liquid connection, the central liquid bridge has already been rapidly stretched and thinned. As the local curvature radius of the liquid bridge continues to decrease, the Laplace pressure difference on both sides of the constricted area increases, further promoting the flow of liquid out of the neck area of the liquid bridge and toward both sides. When the neck radius decreases below the critical scale, capillary instability will cause the liquid bridge to break, resulting in the original droplet splitting into two separate daughter droplets. The two daughter droplets contract under the influence of surface tension and gradually revert to a stable spheroidal shape on the low-adhesion SLIPSs.

**Figure 4:**
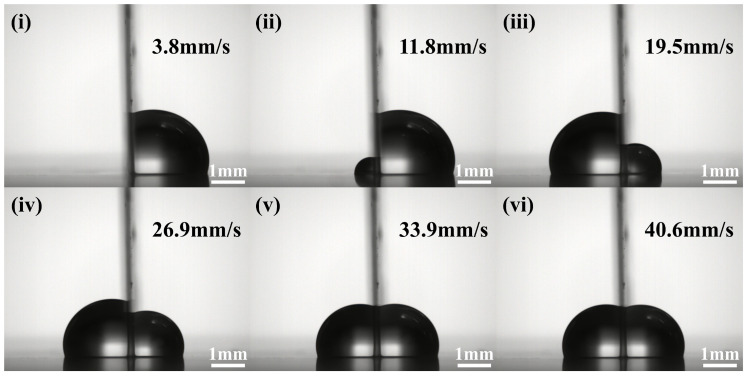
Droplet cutting results at different cutting speeds using the blade with a thickness of 0.1 mm. (**i**) V_CUT_ = 3.8 mm/s; (**ii**) V_CUT_ = 11.8 mm/s; (**iii**) V_CUT_ = 19.5 mm/s; (**iv**) V_CUT_ = 26.9 mm/s; (**v**) V_CUT_ = 33.9 mm/s; (**vi**) V_CUT_ = 40.6 mm/s.

We used the blade with a thickness of 0.1 mm to investigate the effect of V_CUT_ on droplet cutting performance, as shown in Figure 4. When the V_CUT_ is relatively low (V_CUT_ = 3.8 mm/s), the liquid on both sides of the blade has enough time to deform, flow, and redistribute. The liquid on both sides tends to remain in a connected state, eventually leading to the overall migration of the droplets to one side of the blade (Figure 4(i)). As the V_CUT_ increases, the difference in the volumes of the droplets on both sides of the blade gradually decreases (Figure 4(ii–iv)). When the V_CUT_ reaches 33.9 mm/s, the blade can quickly pass through the droplet and form a geometric barrier before the liquid is fully redistributed, achieving approximately symmetrical droplet cutting (Figure 4(v)). When the V_CUT_ was further increased to 40.6 mm/s, the droplets could still achieve stable symmetrical cutting (Figure 4(vi)). Therefore, there is a critical cutting speed (V_CR-CUT_) in the droplet cutting process. Only when the V_CUT_ is higher than this critical value can the blade reliably cut the droplet into two daughter droplets of equal volume.

#### 3.3.2. Effect of Blade Thickness on Droplet Cutting

In this study, we define the minimum speed at which a droplet can be successfully cut in half as the V_CR-CUT_. We use the following criteria to determine whether the droplets have been successfully cut in half: the difference in the projected areas of the two daughter droplets as viewed from the front is less than 3%, and the two daughter droplets can be stably distributed on both sides of the blade. The projected area of the daughter droplets under different cutting speeds was measured using ImageJ. Near the critical value, the V_CUT_ was tested with a step of 1 mm/s.

**Figure 5:**
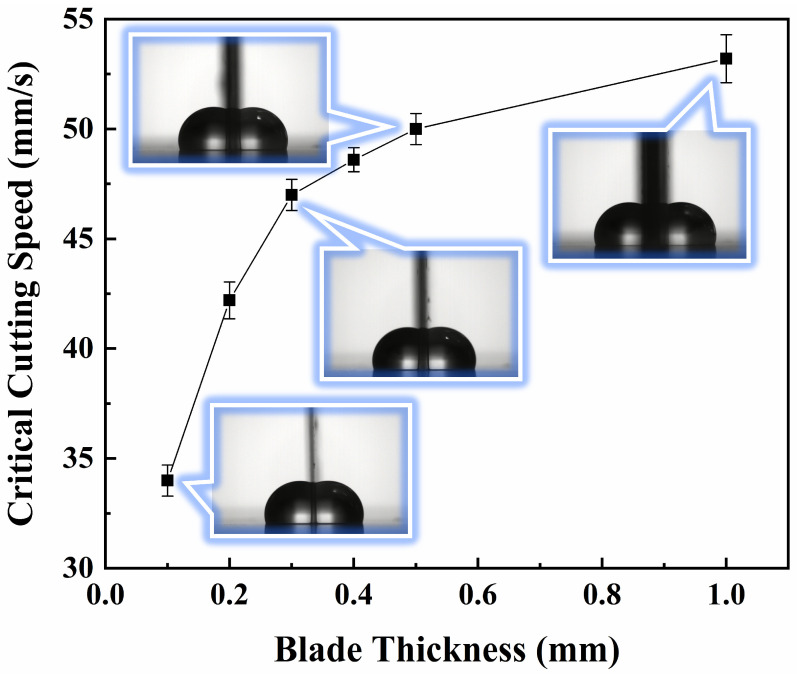
Critical cutting speeds of blades of different thicknesses.

We studied the V_CR-CUT_ under different blade thicknesses, and the results are shown in Figure 5. The V_CR-CUT_ under each condition was measured five times, and the average value was taken as the V_CR-CUT_ for that condition. The error bars in Figure 5 represent the standard deviations of the five groups of data. The cutting results for blades of different thicknesses at the V_CR-CUT_, shown in Figure 5, were captured frame by frame during the cutting process using Adobe Premiere (Pro, 2017). With the increase in blade thickness, V_CR-CUT_ shows a monotonically upward trend. For the blade with a thickness of 0.1 mm, the V_CR-CUT_ is approximately 33.9 mm/s. As the blade thickness increased to 0.2, 0.3, 0.4, and 0.5 mm, the V_CR-CUT_ increased to approximately 42.2, 47.0, 48.5, and 50.1 mm/s, respectively. When the blade thickness was further increased to 1.0 mm, V_CR-CUT_ reached approximately 53.1 mm/s. These results indicate that a thicker blade requires higher V_CUT_ to achieve reliable droplet half-cutting.

In this study, the bottom surface of the blade is flat, and its contact width is equal to the blade thickness. Increasing the blade thickness directly increases the width of the solid barrier introduced into the droplet. Thicker blades will push out a larger volume of liquid from the central area of the droplet and form a wider blade–droplet interaction area. Therefore, before the central liquid bridge is completely depleted, the droplets must undergo a more intense lateral redistribution process. As the blade thickness increases, the cutting process is no longer limited to localized interface disturbances. A thicker blade is more like a wider moving baffle rather than a narrow cutting edge. During the cutting process, the liquid must be displaced over a greater lateral distance and redistributed around a wider solid surface. This will enhance the viscous dissipation of the internal flow of the liquid, increase the contact area between the droplet and the blade surface, and improve the stability of the liquid bridge near the side wall of the blade. Under low-speed cutting conditions, these effects allow the droplet interface to have enough time to adjust its shape around the blade and maintain a connected state, thereby preventing the droplet from splitting in half. Thicker blades require higher cutting speeds to suppress fluid redistribution and force the droplet interface to split rapidly. It is worth noting that when the blade thickness exceeds 0.3 mm, the increase in the V_CR-CUT_ gradually decreases. This suggests that, for thinner blades, the cutting process is more sensitive to blade thickness, as even a slight increase in thickness can significantly alter local droplet deformation and liquid displacement behavior. When the thickness of the blade reaches a certain level, the droplet splitting process is mainly controlled by the overall surface tension of the droplet and the rearrangement of the liquid around the blade. The influence brought by further increasing the thickness of the blade gradually weakens.

Overall, both blade V_CUT_ and blade thickness are key parameters affecting the reliability of blade-assisted droplet cutting on the PCB-SLIPS platform. A sufficiently high V_CUT_ is necessary to overcome liquid flow and asymmetric splitting, while a thinner blade helps reduce the required V_CUT_ and minimize droplet deformation.

### 3.4. EWOD-Assisted Droplet Splitting After Blade Cutting

#### 3.4.1. Mechanism of EWOD-Assisted Droplet Splitting

**Figure 6:**
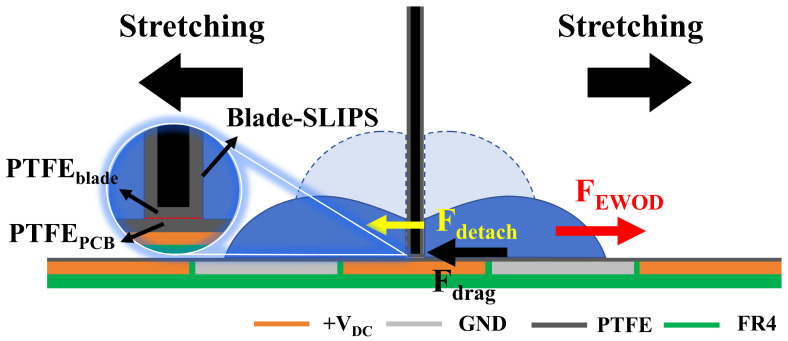
Schematic diagram of force analysis during the droplet splitting process.

After the droplets are self-centered through the PACE trap and cut in half by the blade, the daughter droplets on both sides of the blade meet both the Blade-SLIPS and the PCB-SLIPS. For the convenience of analysis, a force model is established with the right daughter droplet as the research object, and the left daughter droplet can be treated in a symmetrical manner. During the experiment, the aluminum alloy blade was electrically isolated from the linear actuator by an insulated blade holder and remained electrically floating at all times. Therefore, blades are mainly used as mechanical barriers, but they may still cause disturbances to the local electric field. This influence was qualitatively considered in the subsequent discussion of the blade shielding effect. The schematic diagram of the force analysis is shown in Figure 6. Under the electrode configuration where +V_DC_ is applied to the electrode below the blade and the right electrode is grounded, the droplet is subjected to a lateral EWOD driving force to the right. From an energy perspective, the EWOD driving force can be expressed as [29]:

(1) $$F_{EWOD}\left(V,b,x\right)=\frac{1}{2}C_{eff}V^{2}\Lambda_{EW}\left(b,x\right)$$

where C_eff_ is the equivalent capacitance per unit area of PCB-SLIPS, V is the voltage applied to the electrode, b is the thickness of the blade, x is the displacement of the droplet moving outward from the side wall of the blade, and Λ_EW_ is the effective EWOD driving length. The EWOD driving force is related to the square of the applied voltage [30,31]. As the droplet is stretched to the right, it experiences a normal detachment resistance of Blade-SLIPS (F_detach_). The detachment resistance experienced by the droplet during this process primarily includes the Young-Dupré-type effective detachment resistance [32,33], the normal contact line pinning resistance [34], the viscous dissipation of the polydimethylsiloxane wetting ridge [35,36], and oil film/microporous dissipation within the PTFE porous membrane [37,38]. In addition, during the blade cutting process, when the PTFE coating on the bottom surface of the blade just touches the PTFE on the PCB substrate, the blade stops moving downward (see the enlarged illustration at the contact surface in Figure 6). This allows the blade to completely cut the mother droplet and physically split the daughter droplets on both sides of the blade. Although there is no liquid bridge connection between the droplets on either side of the blade, there will be contact line pinning resistance at the lower edge of Blade-SLIPS [39]. Furthermore, since the droplets detach from the Blade-SLIPS due to the EWOD forces driving them, the droplets will also be subject to surface drag (F_drag_) from the PCB-SLIPS. The main forces they must overcome during the movement process are the pinning force at the contact line with the PCB-SLIPS and the viscous drag of the oil film [40,41].

Therefore, the condition for the daughter droplet to be successfully detached from Blade-SLIPS is

(2) $$F_{EWOD}\geq F_{detach}+F_{drag}$$

Substituting Equation (1), we obtain the critical splitting voltage as:

(3) $$V_{cr}\left(b\right)=\underset{0\leq x\leq x_{d}}{\max}\sqrt{\frac{2\left[F_{detach}\left(b,x\right)+F_{drag}\left(b,x\right)\right]}{C_{eff}\Lambda_{EW}\left(b,x\right)}}$$

where $x_{d}$ is the displacement when the sub-droplet is completely detached from the side wall of the blade.

The process of droplet detachment from the blade surface can be divided into four stages: initial adhesion, external pull-off initiation, critical detachment, and rapid detachment. In the initial adhesion stage, the wetting area between the daughter droplet and Blade-SLIPS is the largest, and the blade side wall adhesion force, normal contact line pinning resistance, and local resistance at the lower edge are all at a relatively high level. Therefore, F_detach_ reaches its maximum value. At this point, the effective overlap between the daughter droplet and the grounding electrode has not been fully established, and the F_EWOD_ is relatively low. If the applied voltage is insufficient, the daughter droplet will only undergo localized deformation or slight stretching and will not detach completely from the Blade-SLIPS. During the external pull-off initiation stage, the daughter droplet begins to deform and move outward under F_EWOD_. As the outer side of the daughter droplet gradually covers the grounding electrode, Λ_EW_ increases, and thus F_EWOD_ gradually increases. Meanwhile, the wetting height of the daughter droplet on the Blade-SLIPS begins to decrease, the wetting area reduces, causing F_detach_ to gradually decline. During the critical detachment stage, droplet deformation is most significant. The contact line between the daughter droplet and the Blade-SLIPS undergoes de-pinning, and the detachment contact area rapidly shrinks. This stage is critical to the success of the droplet splitting. On the one hand, F_detach_ continues to decrease rapidly. On the other hand, due to increased deformation at the bottom of the droplet and increased local oil film shear, F_drag_ may exhibit a local peak. During the rapid detachment stage, the primary wetting contact between the daughter droplet and the Blade-SLIPS has largely been broken, and F_detach_ has decreased significantly. At this point, F_EWOD_ is mainly used to overcome F_drag_ and push the daughter droplet to continue moving outward. Due to the rapid weakening of the constraint on the daughter droplet by Blade-SLIPS, the droplets gradually revert to a more stable spheroidal shape. When the daughter droplet has completely detached from the Blade-SLIPS, F_detach_ approaches zero, and the subsequent movement of the droplet is mainly determined by the balance between F_EWOD_ and F_drag_. At this point, the droplet is no longer controlled by the detachment resistance of Blade-SLIPS but behaves as the droplet driving process on the PCB-SLIPS.

#### 3.4.2. Effect of Electrode Shape and Blade Thickness on Droplet Splitting

In this study, we define the minimum voltage at which the daughter droplets on both sides of the blade can be completely detached from Blade -SLIPS as the critical splitting voltage (U_CR_). Since droplet detachment from Blade-SLIPS is subject to complex resistance, minute changes in voltage are not enough to make a significant difference in droplet splitting, so we test with a step of 5 V. In the test results, the lowest voltage at which the daughter droplets successfully split the most times was set as the U_CR_ under this condition.

**Figure 7:**
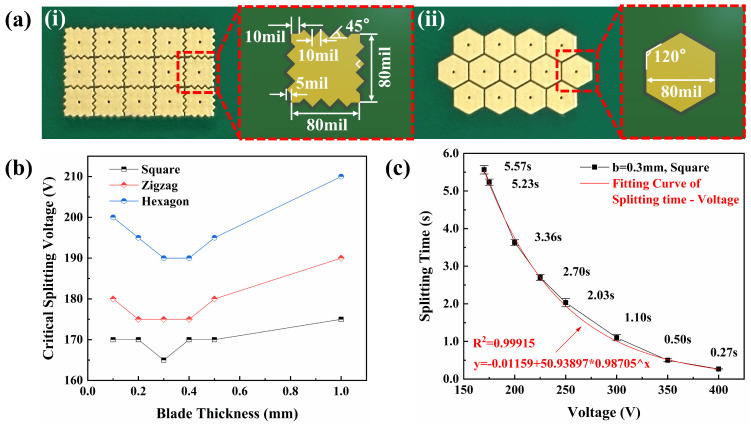
(**a**) Electrode geometric structure and dimensions: (**i**) zigzag, (**ii**) hexagon; (**b**) the relationship between blade thickness and the critical splitting voltage; (**c**) the relationship between the applied voltage and the splitting time for the square electrode with b = 0.3 mm.

We compared the U_CR_ corresponding to three electrode shapes, square, zigzag, and hexagon, at different blade thicknesses with a step of 5 V. The results are shown in Figure 7b. The U_CR_ under each condition was repeatedly tested 10 times. The results of droplet splitting under various voltage conditions were recorded using a CCD camera to serve as the basis for determining the U_CR_. The geometric dimensions of the zigzag and hexagon electrodes are shown in Figure 7a(i,ii). Among the three electrode patterns, the square electrode consistently exhibited the lowest U_CR_. For the square electrode, within the tested blade thickness range, the U_CR_ remains approximately within a narrow range of 165–175 V and reaches its lowest value when the blade thickness is 0.3 mm. In contrast, the zigzag electrode requires a higher voltage, ranging from 175 to 190 V. The U_CR_ of the hexagon electrode is the highest, ranging from 190 to 210 V. These results indicate that the square electrode is more conducive to droplet splitting.

The lower U_CR_ of the square electrode is attributed to its larger effective electrode area and a more direct electric field distribution along the splitting direction [23]. The square electrode has a regular straight-edge boundary, which enables the droplets to form a more effective three-phase contact line overlap with the grounded target electrode, thereby generating a stronger horizontal EWOD force component. In contrast, the zigzag electrode contains inclined and discontinuous edge structures. Although these sharp edges may locally enhance the electric field, part of the EWOD force is distributed in a diagonal direction and does not fully contribute to the horizontal splitting of the droplets [41]. The hexagon electrode has the smallest effective area (approximately 0.87 times that of the square electrode), and the slanted sides of the hexagon electrode further reduce the overlap of the effective contact lines along the horizontal direction. Therefore, the hexagonal electrode generates the smallest net EWOD force at the same voltage [42]. As a result, the zigzag electrode and the hexagon electrode require a higher voltage to overcome the detachment force.

For all three electrode patterns, the U_CR_ initially decreases and then increases as the blade thickness increases. This result indicates that the blade thickness has a dual effect on the U_CR_. On the one hand, appropriately increasing the thickness of the blade can make the daughter droplets on both sides of the blade have a more obvious initial geometric pre-splitting after cutting, which is conducive to the formation of more effective overlap between the droplets and the grounding electrode, increasing the Λ_EW_ and thereby reducing the U_CR_. This is consistent with the findings of Al-Lababidi et al. [23]. On the other hand, as the blade thickness continues to increase, the shielding effect of the metal blade against the local electric field becomes stronger. At the same time, localized pinning at the lower edge of the Blade-SLIPS and increased viscous dissipation in the oil film cause F_detach_ and F_drag_ to increase, thereby leading to a rise in U_CR_. Therefore, there is no simple monotonic relationship between blade thickness and U_CR_, but rather it is determined by the competition between “geometric pre-splitting enhancement” and “blade shielding enhancement”.

It exhibits the best performance under the condition of a square electrode with a blade thickness of 0.3 mm. Under this condition, we compared the droplet splitting times under different splitting voltages (U_SV_). The results are shown in Figure 7c. The splitting time under different voltage conditions was repeated five times. The splitting time was calculated frame by frame using Adobe Premiere (Pro, 2017), and the average of the five measurement results was taken as the splitting time under this voltage. The error bars in Figure 7c represent the standard deviations of the five groups of data. There is a strong exponential relationship between the droplet splitting time and the U_SV_ (see the fitting curve in Figure 7c), with R^2^ = 0.99861. The droplet splitting time decreases exponentially with the increase in the U_SV_. When U_SV_ = U_CR_ = 165 V, the droplet splitting time is 5.57 s. When U_SV_ = 400 V, the droplet splitting time is 0.27 s. The splitting time was shortened by 95.2%, greatly improving the droplet splitting efficiency. We compared the splitting time of blades with thicknesses of 0.1 mm, 0.3 mm, 0.5 mm, and 1 mm at a U_SV_ of 400 V with a square electrode. The results are shown in Video S2. The results are consistent with the trend of the U_CR_ of the square electrode shown in Figure 7c. The splitting time is the shortest when b = 0.3 mm, which is 0.27 s. The splitting times for b = 0.1 mm and 0.5 mm are approximately the same, being 0.47 s and 0.53 s, respectively. The maximum splitting time for b = 1 mm is 0.67 s. The results show that when the applied voltage exceeds the U_CR_, the higher the applied voltage, the shorter the droplet splitting time, which can effectively improve the droplet splitting efficiency.

**Figure 8:**
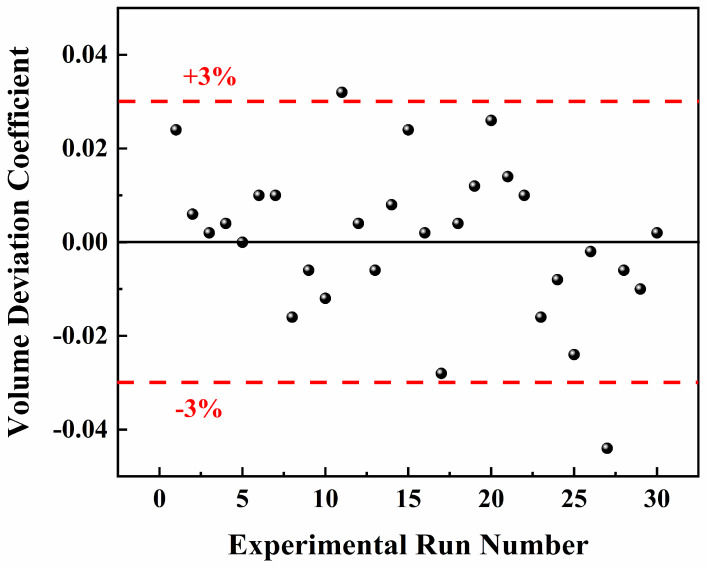
Variation in volume deviation coefficient f over 30 independent experimental runs.

To verify the effectiveness and reliability of the droplet splitting strategy proposed in this paper, we performed a statistical analysis of the volumes of the daughter droplets after splitting. The projected areas of the two daughter droplets after splitting were measured using ImageJ. Let A_L_ and A_R_ be the projected areas of the left and right daughter droplets, respectively. The volume deviation coefficient between the two daughter droplets is f = (A_L_ − A_R_)/5. If f ≤ 3%, it is considered that the droplet has been successfully split in half. Taking a blade with a thickness of 0.3 mm and a U_SV_ of 400 V as an example, 30 consecutive independent droplet splitting experiments were conducted on 10 μL droplets, and the f in these 30 experiments were calculated. The results are shown in Figure 8. Among the 30 independent experiments, f was within 3% in 28 experiments, and f was between 3% and 5% in only two experiments. The success rate of droplet splitting was 93.3%. This indicates that the droplet splitting strategy proposed in this paper offers high accuracy and reliability for the equal-volume cutting of droplets.

In addition, based on the previous experiment, we continued to evaluate the durability of the droplet splitting strategy proposed in this study. A continuous droplet splitting cycle test was conducted on 10 μL droplets using the same PCB-SLIPS chip and the same Blade-SLIPS blade with a thickness of 0.3 mm and a U_SV_ of 400 V. The experimental results show that successful droplet splitting can be achieved with relatively high efficiency during the first 41 cycles of testing. As the cyclic testing continued, performance began to decline before the 53rd cycle, as evidenced by a gradual increase in splitting time. In the 53rd and subsequent cycle tests, the daughter droplets failed to detach from the Blade-SLIPS. The performance was restored after adding silicone oil or replacing the new membrane. Since the linear actuator was used to precisely control the stop position of the blade, no obvious damage was observed in PCB-SLIPS and Blade-SLIPS in all cyclic test experiments. After 41 cycles of testing, the reason for the performance decline and even the inability to achieve droplet splitting was that during the continuous process of droplet self-centering and cutting, the silicone oil in PCB-SLIPS and Blade-SLIPS was squeezed by the droplets, resulting in redistribution and causing uneven local distribution. This increases the viscous resistance on the surface of the chip and the blade, making it difficult for the droplets to split. In severe cases, the resistance is so great that the droplets cannot be split at all. Therefore, under the conditions of this experiment, it can support approximately 50 splitting cycles. The initial splitting performance can be quickly restored by supplementing silicone oil or replacing the membrane with a new one, and the number of cycles for splitting can be increased.

## 4. Conclusions

This study proposes a fully automated, precise droplet splitting method for the open PCB-SLIPS DMF platform in combination with low-adhesion blade-assisted cutting. It provides a simple, low system complexity, and highly controllable method for precise droplet splitting on open DMF platforms through three simple and controllable automated steps: precise positioning via active droplet self-centering, easy-to-maintain low-adhesion blade-assisted cutting, and EWOD-assisted droplet splitting. We achieved active droplet position calibration by using a simple 3 × 3 square electrode array unit in combination with a voltage configuration strategy of high voltage around the outside and a grounded center. The droplet positioning deviation in this method is less than 0.06 mm. Compared with the EWOD free-drive mode, the positioning deviation can be reduced by more than 95% (300 V), which can provide reliable and highly repeatable initial droplet positioning. This study systematically analyzes the effects of blade V_CUT_ and blade thickness on droplet cutting performance. The mechanism of droplet splitting was described in detail, including the dynamic change analysis of the force applied during the droplet splitting process, the determination of the critical splitting conditions, and the influence mechanism of the blade thickness on the U_CR_. The relationship between the blade thickness and the U_CR_ was compared for three different electrode shapes: square, zigzag, and hexagon. The square electrode exhibits the lowest U_CR_. There is no simple monotonic relationship between blade thickness and U_CR_. Both exhibit a trend of first decreasing and then increasing, which is determined by the competition between “geometric pre-splitting enhancement” and “blade shielding enhancement”. Increasing USV can significantly accelerate the droplet splitting process. When the U_SV_ is 400 V, and the blade thickness is 0.3 mm, the droplet splitting time is shortened from 5.57 s under the U_CR_ condition to 0.27 s, reducing the time by 95.2%. The current validation is limited to deionized water. This method offers an accurate, stable, and repeatable droplet splitting strategy with great potential for practical applications. However, its splitting performance still needs to be further validated before it can be applied to actual biological samples.

## Data Availability

The original contributions presented in this study are included in the article. Further inquiries can be directed to the corresponding author.

## Supplementary Materials

The following supporting information can be downloaded at: https://www.mdpi.com/article/10.3390/mi17070839/s1, Video S1: The Complete Automated Splitting Process of a 10 µL Droplet; Video S2: The splitting time of blades with thicknesses of 0.1 mm, 0.3 mm, 0.5 mm, and 1 mm at a splitting voltage of 400 V under a square electrode.

## References

[1] Papamatthaiou S., Menelaou P., Oussallam B.E., Moschou D. (2025). Recent advances in bio-microsystem integration and Lab-on-PCB technology. Microsyst. Nanoeng..

[2] McHale G., Orme B.V., Wells G.G., Ledesma-Aguilar R. (2019). Apparent Contact Angles on Lubricant-Impregnated Surfaces/SLIPS: From Superhydrophobicity to Electrowetting. Langmuir.

[3] Kalyani N., Singh R., Mishra A., Deshpande R.A., Balaman D., Taboryski R., Svendsen W.E. (2025). Enhancing Digital Microfluidics: A Comprehensive Investigation into the Performance of Slippery Liquid-Infused Porous Surfaces. ACS Appl. Mater. Interfaces.

[4] Mahapatra S., Kumari R., Chandra P. (2024). Printed circuit boards: System automation and alternative matrix for biosensing. Trends Biotechnol..

[5] Huang H.Y., Xu C.P., Zhan T., Li S.Y., Han G.C., Zhang B., Cen Y.Y. (2025). Easy-to-Fabricate Digital Microfluidic Chip Based on PCB With Glucose Enzyme-Free Detection Function. IEEE Sens. J..

[6] Yi Z.C., Feng H.Q., Zhou X.F., Shui L.L. (2020). Design of an Open Electrowetting on Dielectric Device Based on Printed Circuit Board by Using a Parafilm M. Front. Phys..

[7] Feng H.Q., Yi Z.C., Yang R.Z., Qin X.F., Shen S.T., Zeng W.J., Shui L.L., Zhou G.F., Zhang C.F. (2020). Designing Splicing Digital Microfluidics Chips Based on Polytetrafluoroethylene Membrane. Micromachines.

[8] Kim H., Chung S.K., Lee J. (2024). PCB-based digital microfluidic platform for droplet mixing on an open surface. Micro Nano Syst. Lett..

[9] Dong C., Jia Y.W., Gao J., Chen T., Mak P.I., Vai M.I., Martins R.P. (2017). A 3D microblade structure for precise and parallel droplet splitting on digital microfluidic chips. Lab Chip.

[10] Guan Y., Tu J.Y., Li B.Y., Fu J.W., Zhu M.N., Chen X.Y., Zhou C. (2020). Stripped Electrode Based Electrowetting-on-Dielectric Digital Microfluidics for Precise and Controllable Parallel Microdrop Generation. Langmuir.

[11] Agnihotri S.N., Raveshi M.R., Nosrati R., Bhardwaj R., Neild A. (2025). Droplet splitting in microfluidics: A review. Phys. Fluids.

[12] Wang Z.L., Bian X.H., Chen L.G. (2020). A Numerical Study of Droplet Splitting using Different Spacers in EWOD Device. BioChip J..

[13] Xing Y.R., Liu Y., Chen R.F., Li Y.Y., Zhang C.Z., Jiang Y.W., Lu Y., Lin B.C., Chen P.Z., Tian R.J. (2021). A robust and scalable active-matrix driven digital microfluidic platform based on printed-circuit board technology. Lab Chip.

[14] Bormashenko E., Bormashenko Y. (2011). Non-Stick Droplet Surgery with a Superhydrophobic Scalpel. Langmuir.

[15] Yanashima R., García A.A., Aldridge J., Weiss N., Hayes M.A., Andrews J.H. (2012). Cutting a Drop of Water Pinned by Wire Loops Using a Superhydrophobic Surface and Knife. PLoS ONE.

[16] Kiani M.J., Dehghan A., Saadatbakhsh M., Asl S.J., Nouri N.M., Pishbin E. (2022). Robotic digital microfluidics: A droplet-based total analysis system. Lab Chip.

[17] Han X., Tan S.D., Jin R.Y., Jiang L., Heng L.P. (2023). Noncontact Charge Shielding Knife for Liquid Microfluidics. J. Am. Chem. Soc..

[18] Sagar N., Bansal S., Sen P. (2022). Open-Chip Droplet Splitting in Electrowetting. Adv. Mater. Interfaces.

[19] Chen C.H., Yang K.H. (2025). Efficient in-droplet concentration of particles for digital microfluidics on coplanar electrodes by integrating electrowetting-on-dielectric (EWOD) and surface acoustic wave (SAW) technologies. Sens. Actuator B Chem..

[20] He X.D., Zhang J.F., Zhang X.P., Deng Y.Q. (2019). Droplet manipulation with polarity-dependent low-voltage electrowetting on an open slippery liquid infused porous surface. Soft Matter.

[21] Fan S.K., Yang H.P., Wang T.T., Hsu W. (2007). Asymmetric electrowetting—Moving droplets by a square wave. Lab Chip.

[22] He X.D., Xu J.S., Yang B., Yang F.L. (2023). Asymmetric electrodes for droplet directional actuation by a square wave on an open surface. Results Phys..

[23] Al-Lababidi M., Abdelgawad M. (2023). Minimum movable droplet volume in digital microfluidics depends on the grounding scheme in addition to electrode size. Sens. Actuator A Phys..

[24] Alistar M., Gaudenz U. (2017). Opendrop: An integrated do-it-yourself platform for personal use of biochips. Bioengineering.

[25] Abdelgawad M., Park P., Wheeler A.R. (2009). Optimization of device geometry in single-plate digital microfluidics. J. Appl. Phys..

[26] Yang X.L., Liu X., Hess D.W., Breedveld V. (2017). Underwater Oil Droplet Splitting on a Patterned Template. Langmuir.

[27] Xing S., Harake R.S., Pan T. (2011). Droplet-driven transports on superhydrophobic-patterned surface microfluidics. Lab Chip.

[28] Melin J., van der Wijngaart W., Stemme G. (2005). Behaviour and design considerations for continuous flow closed-open-closed liquid microchannels. Lab Chip.

[29] Wu X.Y., Tang D.B., He Q.P., Liu L.X., Jia Z.Y., Tan Y.Y. (2023). Research progress of electrode shapes in EWOD-based digital microfluidics. RSC Adv..

[30] Yamamoto K., Takagi S., Ichikawa Y., Motosuke M. (2022). Lubrication effects on droplet manipulation by electrowetting-on-dielectric (EWOD). J. Appl. Phys..

[31] Vo Q., Tran T. (2021). Dynamics of droplets under electrowetting effect with voltages exceeding the contact angle saturation threshold. J. Fluid Mech..

[32] Daniel D., Koh X.Q. (2023). Droplet detachment force and its relation to Young-Dupre adhesion. Soft Matter.

[33] Sadullah M.S., Xu Y.F., Arunachalam S., Mishra H. (2024). Predicting droplet detachment force: Young-Dupré Model Fails, Young-Laplace Model Prevails. Commun. Phys..

[34] Sadullah M.S., Panter J.R., Kusumaatmaja H. (2020). Factors controlling the pinning force of liquid droplets on liquid infused surfaces. Soft Matter.

[35] Naga A., Rennick M., Hauer L., Wong W.S.Y., Sharifi-Aghili A., Vollmer D., Kusumaatmaja H. (2024). Direct visualization of viscous dissipation and wetting ridge geometry on lubricant-infused surfaces. Commun. Phys..

[36] Zhang J.Q., Wang X.J., Wang Z.Y., Pan S.F., Yi B., Ai L.Q., Gao J., Mugele F., Yao X. (2021). Wetting ridge assisted programmed magnetic actuation of droplets on ferrofluid-infused surface. Nat. Commun..

[37] He X.D., Yang B., Li J., Zhang X.P., Deng Y.Q. (2022). Experimental and theoretical study of electrowetting dynamics on slippery lubricant-infused porous surfaces. Sens. Actuator A-Phys..

[38] Villegas M., Zhang Y.X., Abu Jarad N., Soleymani L., Didar T.F. (2019). Liquid-Infused Surfaces: A Review of Theory, Design, and Applications. ACS Nano.

[39] Xu H.B., Zhou Y.M., Daniel D., Herzog J., Wang X.G., Sick V., Adera S. (2023). Droplet attraction and coalescence mechanism on textured oil-impregnated surfaces. Nat. Commun..

[40] Vega-Sánchez C., Peppou-Chapman S., Zhu L.W., Neto C. (2022). Nanobubbles explain the large slip observed on lubricant-infused surfaces. Nat. Commun..

[41] Jain V., Devarasetty V., Patrikar R. (2017). Effect of electrode geometry on droplet velocity in open EWOD based device for digital microfluidics applications. J. Electrost..

[42] Geng H.Y., Cho S.K. (2019). Antifouling digital microfluidics using lubricant infused porous film. Lab Chip.

